# Molecular and Morphological Differentiation of Common Dolphins (*Delphinus* sp.) in the Southwestern Atlantic: Testing the Two Species Hypothesis in Sympatry

**DOI:** 10.1371/journal.pone.0140251

**Published:** 2015-11-11

**Authors:** Haydée A. Cunha, Rocio Loizaga de Castro, Eduardo R. Secchi, Enrique A. Crespo, José Lailson-Brito, Alexandre F. Azevedo, Cristiano Lazoski, Antonio M. Solé-Cava

**Affiliations:** 1 Laboratório de Mamíferos Aquáticos e Bioindicadores (MAQUA), Faculdade de Oceanografia, Universidade do Estado do Rio de Janeiro, Rio de Janeiro, Rio de Janeiro, Brazil; 2 Laboratorio de Mamíferos Marinos, Centro Nacional Patagónico–CONICET, Puerto Madryn, Chubut, Argentina; 3 Laboratório de Ecologia e Conservação da Megafauna Marinha (EcoMega), Instituto de Oceanografia, Fundação Universitária do Rio Grande, Rio Grande, Rio Grande do Sul, Brazil; 4 Laboratório de Biodiversidade Molecular, Instituto de Biologia, Universidade Federal do Rio de Janeiro, Rio de Janeiro, Rio de Janeiro, Brazil; Smithsonian Institution, UNITED STATES

## Abstract

The taxonomy of common dolphins (*Delphinus* sp.) has always been controversial, with over twenty described species since the original description of the type species of the genus (*Delphinus delphis* Linnaeus, 1758). Two species and four subspecies are currently accepted, but recent molecular data have challenged this view. In this study we investigated the molecular taxonomy of common dolphins through analyses of cytochrome b sequences of 297 individuals from most of their distribution. We included 37 novel sequences from the Southwestern Atlantic Ocean, a region where the short- and long-beaked morphotypes occur in sympatry, but which had not been well sampled before. Skulls of individuals from the Southwestern Atlantic were measured to test the validity of the rostral index as a diagnostic character and confirmed the presence of the two morphotypes in our genetic sample. Our genetic results show that all common dolphins in the Atlantic Ocean belong to a single species, *Delphinus delphis*. According to genetic data, the species *Delphinus capensis* is invalid. Long-beaked common dolphins from the Northeastern Pacific Ocean may constitute a different species. Our conclusions prompt the need for revision of currently accepted common dolphin species and subspecies and of *Delphinus delphis* distribution.

## Introduction

*Delphinus delphis* Linnaeus, 1758 is the earliest dolphin species described that is still valid today. Interestingly, it is one of the species described before the binomial classification system adopted by Linnaeus in the 10^th^ edition of Systema Naturae [1]. The description provided by Linnaeus was originally made by Artedi [2], who, in turn, had recognised and synonymised previous names for the species. The name *delphis*, cited by him in Greek characters, may trace back to Aristotle [2].

In his Systema Naturae, Linnaeus described twelve cetacean species, distributed in four genera. *Delphinus delphis* is the type of a genus that would encompass most of the toothed cetacean species before description of other genera and reallocation of several species to them. After excluding those reallocations, over twenty of those names, described between 1758 and 2002, still corresponded to common dolphin species and subspecies [3–5], but several of those have been synonymised or considered *nomen dubium* [3,5,6]. Until recently, some authors acknowledged a single common dolphin species worldwide (e.g. [7,8]), while others accepted up to three (e.g.[9,10]). This taxonomic uncertainty emerged from an impressive amount of morphological variability in the shape, size and coloration of common dolphins around the world, coupled with a long lasting lack of studies over a large and geographically comprehensive collection of specimens [3,6].

The turning point in *Delphinus* taxonomy happened in 1994, when morphological and genetic analyses provided evidence that common dolphins in the Northeastern Pacific Ocean belonged to two different species living in sympatry [6,11]. They were classified as the short-beaked common dolphin *D*. *delphis* and the long-beaked common dolphin *D*. *capensis* Gray, 1828 [6]. Although a long-beaked species of common dolphin, *Delphinus bairdii* Dall, 1873, had been described for California, Heyning & Perrin [6] followed the conclusions of earlier authors [12] that considered it to be a junior synonym of *D*. *capensis*. As the species *D*. *delphis* and *D*. *capensis* corresponded to the previously known short and long-beaked morphotypes and, as short and long-beaked common dolphins were known to occur in other regions of the world, Heyning & Perrin [6] suggested that the morphological diagnoses proposed for the Northeastern Pacific would hold true worldwide, and that the rostral index (the ratio between rostrum length, RL, and zygomatic width, ZW) would be diagnostic for species identification.

Since then, morphological analyses of common dolphins from different parts of the world used a 1.52 RL/ZW threshold to discriminate between *D*. *delphis* and *D*. *capensis*. Based on this criterion, *D*. *delphis* is considered to occur in the Atlantic and Pacific oceans and in the Mediterranean Sea, including oceanic areas, while *D*. *capensis* would have a patchy coastal distribution, occurring in the Northeastern Pacific (20°N to 40°N), Southeastern Pacific (20°S to 0°), Japan, Southwestern Atlantic (20° to 40°S), Southeastern Atlantic (10°S to ~35°N, and South Africa), the Caribbean coast of Venezuela and possibly the Indian Ocean [7,13,14]. Many authors also accept the subspecies *D*. *delphis ponticus* (dwarf common dolphins from the Black Sea) [7,13,15] and *D*. *capensis tropicalis* (extremely long-beaked common dolphins from the Indian Ocean) [5,7,13].

In spite of the widespread usage of the binomials *D*. *capensis* for long-beaked and *D*. *delphis* for short-beaked common dolphins, many morphological studies around the globe have provided evidence at odds with Heyning & Perrin’s proposal. Amaha [15] analysed 60 measures from 289 skulls of *Delphinus* from most of the genus distribution. She concluded that, outside the Northeastern Pacific, morphological forms could not be clearly assigned to either of the two species, but that the *tropicalis-*form of the Indian Ocean should be considered a third species (*D*. *tropicalis*). In Australia, Bell *et al*. [16] observed that the rostral ratio was not helpful for *Delphinus* species identification because individuals spanned the entire range of RL/ZW values reported by Heyning & Perrin [6]. In South Africa, Saamai *et al*. [17] verified that the rostral ratio of most common dolphins were above the 1.52 rostral ratio threshold, but those dolphins had lower vertebral count compared to long-beaked common dolphins from California. Common dolphins from the Northeastern Atlantic were identified as *D*. *delphis* but also presented several measures, including RL/ZW, that overlapped with those described for short and long-beaked common dolphins from the Northeastern Pacific [18]. Westgate [19] analysed *Delphinus* skulls from both sides of the North Atlantic and concluded that they belonged to *D*. *delphis*, and that the rostral index, as proposed by Heyning & Perrin [6] was insufficient for species identification due to large variance in samples from outside California. In the Southwestern Atlantic, Tavares *et al*. [14] also noticed that the rostral index probably was not diagnostic for species identification, due to its very large variance. Despite so, they suggested that both *D*. *delphis* and *D*. *capensis* occurred in the region.

Genetic studies have also produced evidence against the existence of two species of *Delphinus* worldwide. For example, an early study using cytochrome b sequences found *D*. *delphis* to be paraphyletic in relation to *D*. *capensis* [20], and common dolphins from South Africa (type locality of *D*. *capensis*) were genetically indistinguishable from *D*. *delphis* [21,22]. However, against all evidence, the authors have preferred to consider the two species valid, resorting to *ad hoc* explanations like incomplete lineage sorting and hybridisation, although the latter authors recognised that *D*. *capensis* may prove to be invalid.

The Southwestern Atlantic is one of the few regions where the short and long-beaked morphotypes occur sympatrically [14]. As such, it represents an opportunity to test the hypothesis of two common dolphin species through genetic and morphological analyses. Previous genetic studies [21,22] have analysed only a small sample of common dolphins from the Southwestern Atlantic, without a proper morphological assignment of specimens, and none of those works concluded explicitly about the taxonomy of *Delphinus* in the region. Morphological analyses, on the other hand, have included many specimens and suggested the presence of both *D*. *delphis* and *D*. *capensis* in the Southwestern Atlantic [14,23].

In this study, we analysed a large, geographically comprehensive data set comprising full cytochrome b sequences of all common dolphin morphotypes. More importantly, we included 37 new sequences of short and long-beaked common dolphins from the Southwestern Atlantic. Skulls were examined to determine the morphotype of individuals. Our phylogenetic analyses do not support the existence of more than a single common dolphin species in the Atlantic. On a broader context, genetic data refute the validity of *D*. *capensis*, but do not reject the specific status of long-beaked common dolphins from the Northeastern Pacific.

## Material and Methods

### Tissue sampling

**Fig 1:**
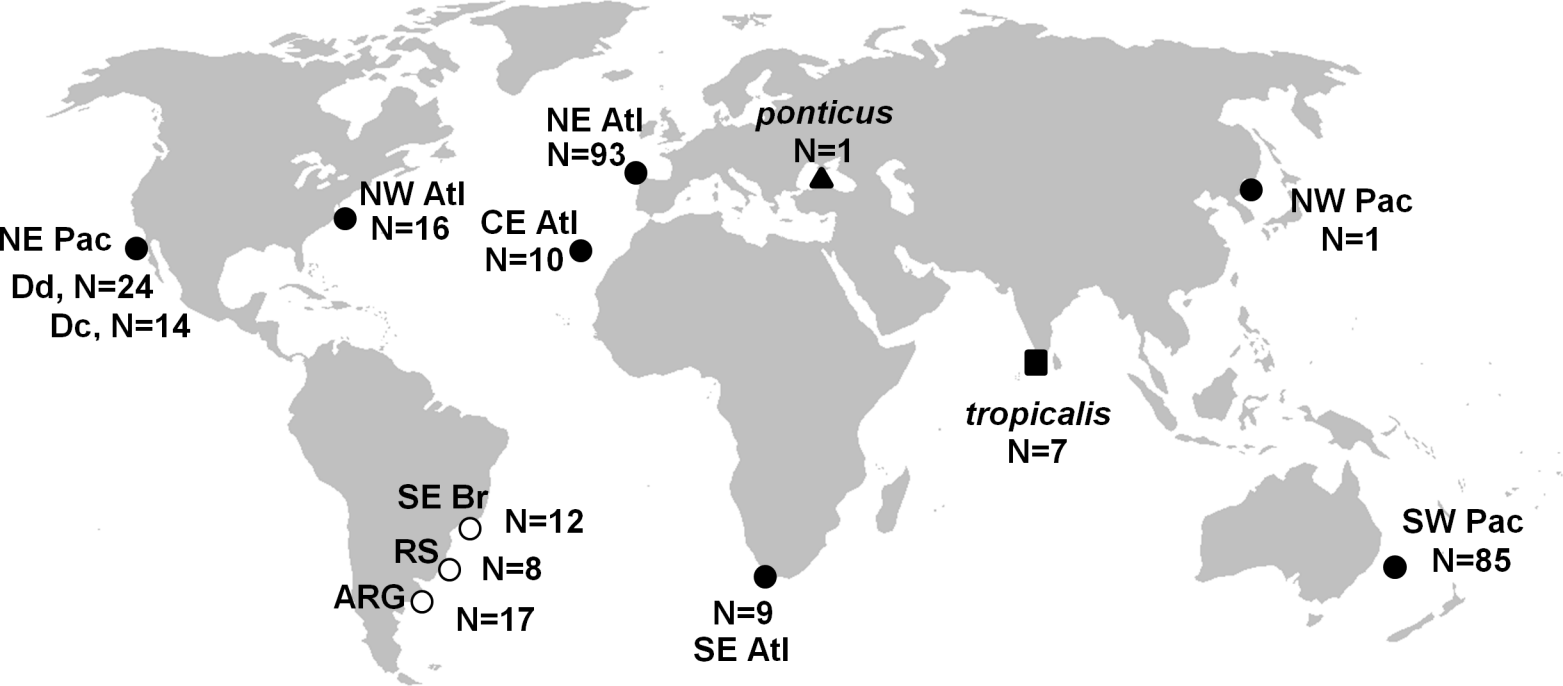
Sampling of common dolphins for this study. White circles indicate new samples; black symbols refer to sequences from GenBank. Sample size is shown between parentheses. The square indicates sequences of the *tropicalis*-form and the triangle the only sequence available of the putative *D*. *d*. *ponticus*. SE Br: Southeastern Brazil (grouping samples from Rio de Janeiro, RJ and São Paulo, SP); RS: Rio Grande do Sul; ARG: Argentina; NW Atl: Northwestern Atlantic; NE Atl: Northeastern Atlantic; CE Atlantic: Central Eastern Atlantic; NE Pac: Northeastern Pacific; SW Pac: Southwestern Pacific. Dc: long-beaked common dolphins “*Delphinus capensis*”; Dd: short-beaked common dolphins *D*. *delphis*.

Tissue samples were collected from stranded or by-caught individuals (N = 29), or through biopsy darting [24] (N = 8), in three areas in the Southwestern Atlantic (SW Atlantic, Fig 1). Sampling permits were issued by the Brazilian Environmental Agency IBAMA/MMA (Instituto Brasileiro do Meio Ambiente e Recursos Renováveis; sampling permits 11495–2 and 16586–2) and the Argentine Environmental Agency (Secretaría de Recursos Naturales y Desarrollo Sustentable de la República Argentina; permit n°006/99). Samples were preserved either in NaCl saturated 20% DMSO solution or in ethanol.

**Fig 2:**
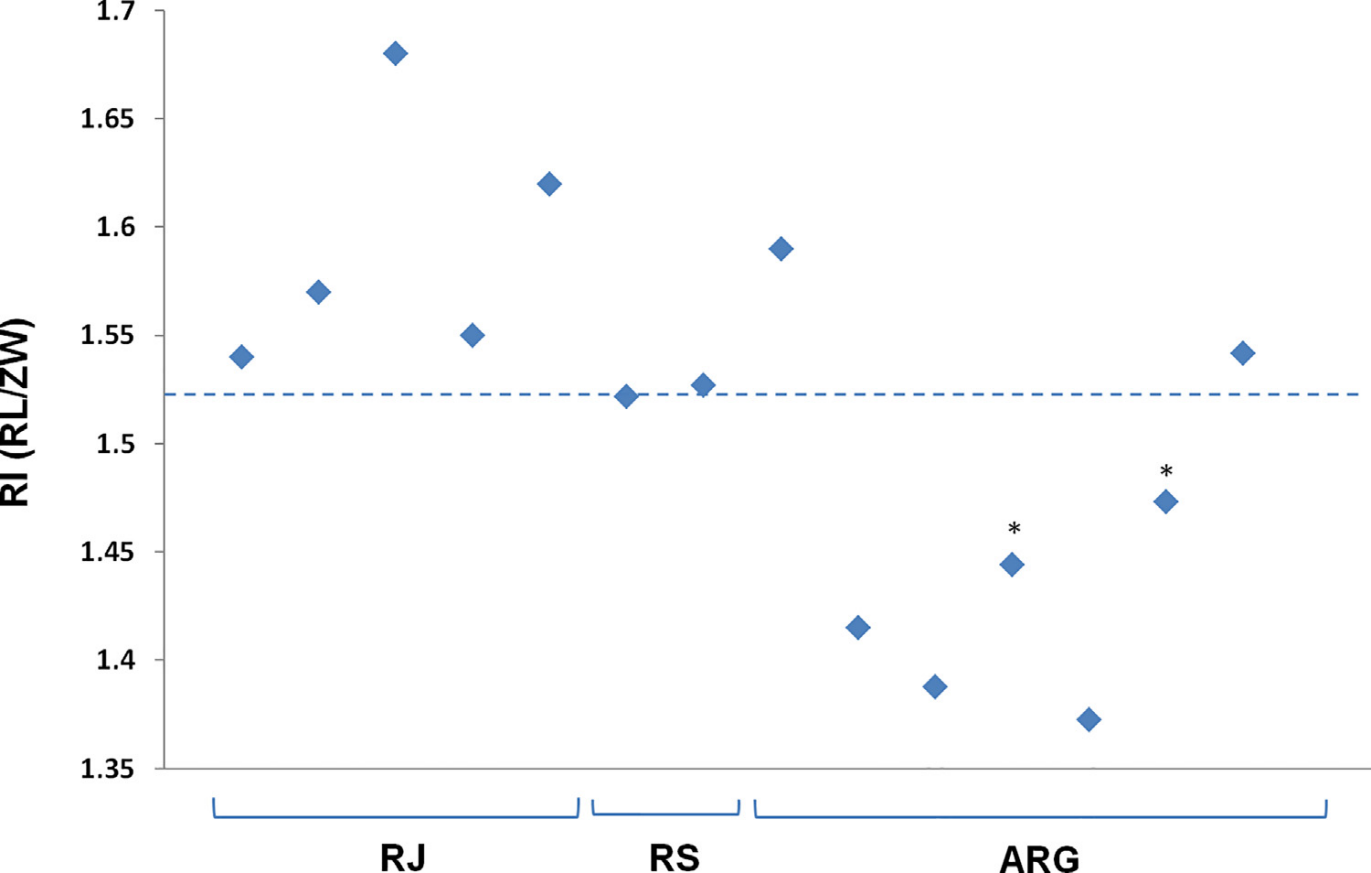
Rostral index (IR). Distribution of IR values of the measured specimens (N = 14). The line indicates the diagnostic threshold proposed by Heyning & Perrin [6]. The two specimens marked with an asterisk were considered immature (see Table 1).

In order to determine the morphotype of individuals from the SW Atlantic, craniometrical measures from 14 of the stranded/by-caught specimens (N = 29) were analysed. Skulls of ten individuals were lost, and the remaining individuals were calves. Specimens were judged mature by fulfilling two criteria: degree of suture of cranial bones and closure of alveoli, and total body length above 190 cm. Following Heyning & Perrin [6], rostral length and zygomatic width were measured and their ratio calculated. Skull measures of the individuals sampled in Argentina (ARG) were available from González [25]. According to the rostral index, all Rio de Janeiro (RJ) and Rio Grande do Sul (RS) samples would be considered *Delphinus capensis*, while ARG samples would belong to *D*. *capensis* (two individuals) and *D*. *delphis* (five individuals) (Table 1, Fig 2). Due to the existence of sexual dimorphism, morphological assignments were further confirmed for males and females by comparing the cranial measurements taken from SW Atlantic for this study to those presented in Tables 5 and 6 of Heyning & Perrin [6].

**Table 1:** Collection and morphological data of the specimens genetically analysed. Specimens marked with an asterisk were considered immature.

Area	Locality	Collection year	Specimen code	Sex	RL/ZW	Observation
RJ	Maricá	2001	MQ152	F	1.54	
	Rio de Janeiro	2003	MQ186	M	1.57	
	Rio de Janeiro	2003	MQ187	M	1.68	
	Rio de Janeiro	2004	MQ189	F	1.55	
	Niterói	2005	MQ204	F	1.62	
	Rio de Janeiro	2011	MQ370		-	Calf
	Saquarema		SAQ1		-	Skull lost
	Off Cabo Frio	2011	Trin01		-	Biopsy sample
	Off Cabo Frio	2011	Trin02		-	Biopsy sample
	Off Cabo Frio	2011	Trin03		-	Biopsy sample
SP	Santos	2004	BP86		-	Calf
RS	Rio Grande	2008	RS1		-	Skull lost
	Rio Grande	2008	RS2	F	1.52	
	Rio Grande	2009	RS3	M	1.53	
	Off Rio Grande	2010	RS4		-	Biopsy sample
	Off Rio Grande	2010	RS5		-	Biopsy sample
	Off Rio Grande	2010	RS6		-	Biopsy sample
	Off Rio Grande	2010	RS7		-	Biopsy sample
	Off Rio Grande	2010	RS8		-	Biopsy sample
	Off Rio Grande	2010	RS10		-	Biopsy sample
ARG	Mar Salvaje	1999	Dd002		-	Calf
	Mar Salvaje	1999	Dd003		-	Skull lost
	Mar Salvaje	1999	Dd004	M	1.59	
	Mar Salvaje	1999	Dd005		-	Skull lost
	Mar Salvaje	1999	Dd006		-	Skull lost
	Mar Salvaje	1999	Dd007		-	Skull lost
	Mar Salvaje	1999	Dd008		-	Skull lost
	Mar Salvaje	1999	Dd010	M	1.42	
	Mar Salvaje	1999	Dd011	F	1.39	
	Mar Salvaje	1999	Dd012*	M	1.44	
	Mar Salvaje	1999	Dd013	F	1.37	
	Mar Salvaje	1999	Dd014		-	Skull lost
	Mar Salvaje	1999	Dd015		-	Skull lost
	Mar Salvaje	1999	Dd016		-	Skull lost
	Mar Salvaje	1999	Dd017*	F	1.47	
	Mar Salvaje	1999	Dd018	M	1.54	
	Mar Salvaje	1999	Dd019		-	Calf

Osteological material from stranded and by-caught specimens are deposited in the following institutions: RJ–Laboratório de Mamíferos Aquáticos e Bioindicadores, Universidade do Estado do Rio de Janeiro; SP–Projeto Biopesca; RS–Laboratório de Ecologia e Conservação da Megafauna Marinha, Fundação Universitária do Rio Grande; ARG–Universidad Nacional de Mar del Plata.

### Genetic analyses

DNA was extracted using the standard phenol-chloroform protocol [26] or DNeasy Blood and Tissue kit (Qiagen). The full mitochondrial cytochrome b gene was PCR-amplified using primers L14724 [27] and an unnamed primer designed by Le Duc *et al*. [20](ccttttccggtttacaagac), in 20μL reactions containing 1U Taq, 200μM dNTP, 2.5mM MgCl_2_, 1μg/μL BSA and 0.5μM of each primer. Amplification thermal conditions were as follows: 3 min at 93°C, 30 cycles of 1 min at 92°C, 1 min at 50°C and 1 min at 72°C, and 5 min of final extension at 72°C.

PCR products were purified using the Illustra GFX PCR DNA and Gel Band Purification Kit (GE) and both strands were sequenced in an ABI3500 using BigDye Terminator v. 3.1 chemistry (Applied Biosystems) with the same primers used for amplification. Sequences were edited in SeqMan 7 (DNAStar Inc.) and deposited in GenBank under accession numbers KM225661-225673.

Cytochrome b sequences were aligned with other 260 common dolphin sequences available in GenBank, including samples from the Atlantic (Northwestern, NW Atl; Northeastern, NE Atl; Central Eastern, CE Atl; and Southeastern, SE Atl) and the Pacific (Northeastern, NE Pac; and Southwestern, SW Pac) oceans (Fig 1, Table 2). Haplotype definition was done in DnaSP [28]. Ten sequences from the SW Pac deposited in GenBank were identified as duplicates by their field codes, and therefore one sequence of each duplicated pair was not included in the analyses. For phylogenetic analyses only haplotype sequences were used (S1 Table). Complete cytochrome b sequences from all other delphinid genera and most species were also used in phylogenetic analyses. Sequences were manually aligned in the software MEGA 5 [29].

**Table 2:** Common dolphin cytochrome b sequences used in this study. Sequences from other studies were obtained from GenBank. Except where noted and in the SE Atl, all specimens had or were assumed as the short-beaked morphotype. Samples from the SW Atl were assigned to morphotypes after skull measurements (Table 1).

Sample locality	Number of sequences	Reference
Northeastern Pacific		
Dc (long-beaked)	12	[22]
	2	[20]
Dd (short-beaked)	23	[22]
	1	[20]
Southwestern Pacific	85	[22]
Northwestern Pacific	1	[30]
Northwestern Atlantic	16	[22]
Central Eastern Atlantic	10	[22]
Northeastern Atlantic	67	[31]
	26	[22]
Southeastern Atlantic	9	[22]
Southwestern Atlantic		
Long-beaked	9	This study
Short-beaked	5	This study
Unknown	23	This study
Indian Ocean (*tropicalis*-form)	1	[20]
	6	[22]
Black Sea (*ponticus*-form)	1	[20]

We used three methods of phylogenetic inference. A Neighbor-Joining tree of cytochrome b haplotypes was built in MEGA using K2P distance, and 10,000 bootstrap replicates were conducted to assess node confidence. The software jModelTest [32] was used to select the most likely model of evolution for Maximum-Likelihood (ML) and Bayesian phylogenetic analyses, which were conducted in PhyML 3.0 [33] and BEAST 1.7.5 [34], respectively. Under both the AIC and BIC criteria, the HKY+I+G model was selected. The ML tree search was performed by the SPR algorithm and the aLRT statistic [35] was used to evaluate node confidence. Bayesian trees were generated using a Yule speciation process. One hundred million MCMC steps were run, from which 10,000 trees were recorded. After verification that all tree parameters had ESS > 200, the MCC search algorithm in TreeAnnotator [34] was used to find the best supported tree. The first 1,000 trees were regarded as ‘burn in’ and discarded. ML and Bayesian trees were visualised using FigTree 1.4 (*http*:*//tree*.*bio*.*ed*.*ac*.*uk/software/figtree/*).

## Results

### Genetic variability

The full common dolphin cytochrome b dataset (297 sequences; 1,140bp) revealed 154 haplotypes, with haplotype and nucleotide diversities of 0.973 and 0.006, respectively. Nineteen cytochrome b haplotypes were found for the SW Atlantic, 18 of them being new haplotypes for this region.

### Phylogenetic analyses

**Fig 3:**
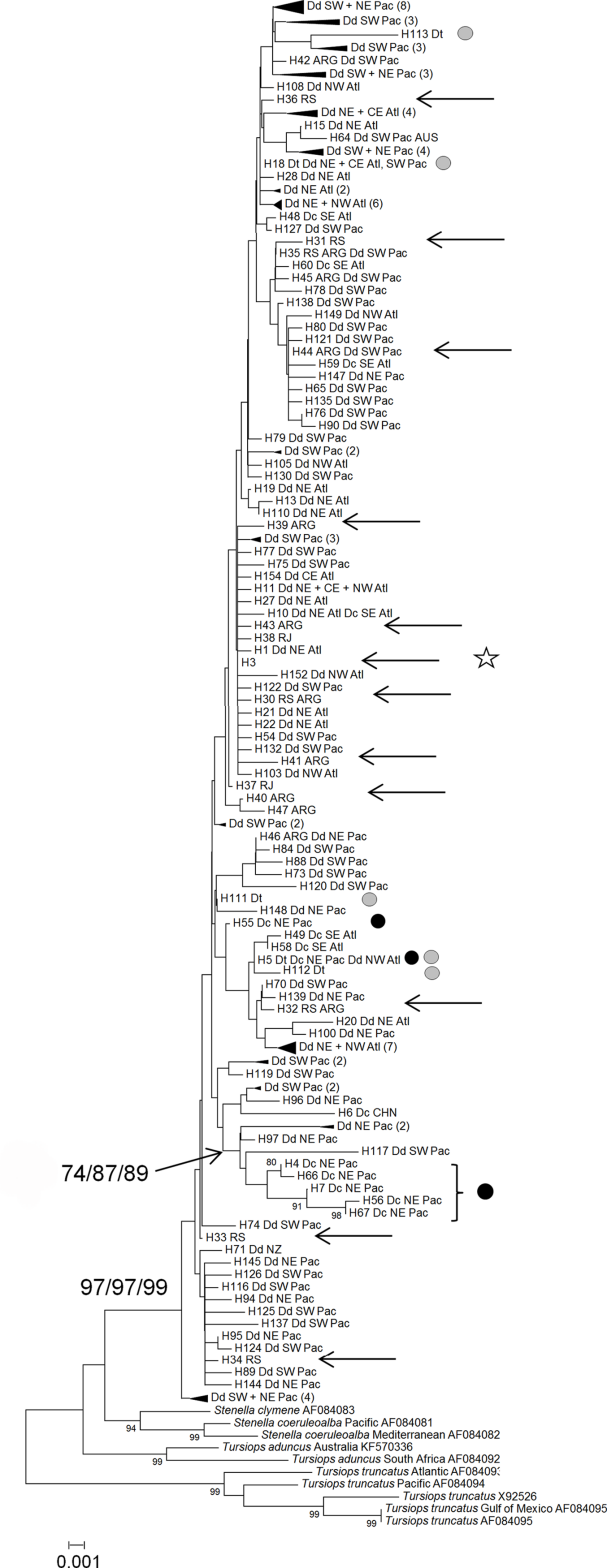
Phylogenetic tree (NJ/ML/BI) of common dolphin cytochrome b haplotypes. Values above nodes correspond to bootstrap (NJ), aLRT (ML) and posterior probability (BI), respectively, > 50%. Arrows indicate sequences generated in this study (SW Atlantic): specimens coded with RJ, RS ARG4 and ARG18 had the long-beaked morphotype, and the remaining specimens coded with ARG had the short-beaked haplotype (see Table 1). Grey circles indicate sequences of the *tropicalis*-form. The star indicates the haplotype shared by short and long-beaked common dolphins (from almost all localities, including the SW Atlantic) and the putative *D*. *d*. *ponticus* (Black Sea). Black circles mark the long-beaked morphotype from the NE Pacific (please note the paraphyly).

The three phylogenetic methods recovered similar topologies. Phylogenetic reconstructions showed genus *Delphinus* as monophyletic (97/97/99; bootstrap, aLRT and PP, respectively), but its sister lineage could not be identified due to lack of resolution (S1 Fig). We used as outgroups for the analyses ten sequences from four species that are closely related to *Delphinus*: *Stenella clymene*, *S*. *coeruleoalba*, *Tursiops aduncus* and *T*. *truncatus* (Fig 3).

Sequences from the three *Delphinus* morphotypes (short-beaked, long-beaked and *tropicalis*-form) appear scattered in the trees, regardless of geographic location. The sequence from the Black Sea is identical to the most common *Delphinus* haplotype. The only clade that appeared consistently, although with low support in NJ and ML analyses, was the one that grouped most of the long-beaked common dolphins from the NE Pacific (Dc NE Pac, 74/87/99, Fig 3).

## Discussion

The main conclusions of this work are that the separation of *Delphinus* species based on the rostral index alone is not justified, and that *D*. *capensis* is not a valid species. Short and long-beaked common dolphins in the Atlantic and in some areas of the Pacific, therefore, all belong to a single species, *Delphinus delphis*. This conclusion supports accumulating evidence from genetic, morphological, ecological and stable isotope data [14–22,36]. We have also found evidence for the existence of an endemic common dolphin species in the NE Pacific, previously proposed by Heyning & Perrin [1] and Rosel *et al*. [11], possibly *Delphinus bairdii* (see discussion below).

### Molecular taxonomy

Our results have several taxonomic implications. The most obvious one is the existence of a single *Delphinus* species in the Atlantic, as individuals morphologically assigned to *D*. *capensis* based on rostral length/zygomatic width (RL/ZW) ratios do not differ genetically from short-beaked individuals from several localities in the South and North Atlantic. That conclusion is reinforced by the fact that samples from the type locality of *D*. *capensis* (South Africa, SE Atl) were included in the analyses. Thus, according to genetic data, *D*. *capensis* is not a valid species. Common dolphins in the Atlantic belong to a single species, *Delphinus delphis* Linnaeus, 1758 (type locality “Oceano Europaeo”, or NE Atlantic). Throughout the Discussion, unless otherwise stated, the terms “short” and “long-beaked” common dolphins refer to morphotypes, not species.

We believe that the main confusion in the taxonomy of *Delphinus* was the strong weight given to rostral length, after the very thorough work of Heyning & Perrin [6]. The existence of two Californian common dolphin species with different rostral index ranges had been previously proposed by Banks & Brownell [37], after analyses of 64 skulls. Heyning & Perrin [6] analysed 26 cranial and 38 body measurements, as well as 19 post-cranial meristics and coloration patterns of a very large number of specimens from California. They did not find significant differences for any of the characters measured, other than coloration patterns and RL/ZW. Since coloration is too variable and not always available for museum specimens, RL/ZW became the rule of thumb for diagnosing the two species in California. Heyning & Perrin [6] correctly concluded that the long-beaked and short-beaked forms belonged to different species, commenting that their conclusion was also supported by genetic data, then in press, by Rosel *et al*. [11], and assigned the long-beaked individuals to *D*. *capensis*, because it had priority over *D*. *bairdii* Dall, 1873, a species with the long-beaked morphotype whose type locality was California, but which had been synonymised with *D*. *capensis* (by van Bree & Purves [12]). Our results completely agree with those of Heyning & Perrin [6] and also with Rosel *et al*. [11] and Kingston & Rosel [38] in that two *Delphinus* species occur in the NE Pacific, off the Californian coast. However, our data do not support Heyning & Perrin’s conclusion that the long-beaked species from California was the same as the long-beaked *Delphinus capensis* from the SE Atlantic. It is clear that even though RL/ZW is significantly different between the two Californian species, it cannot be treated as a diagnostic character between *Delphinus* species worldwide. The large weight put upon the RL/ZW ratio was understandable considering that it was the only significant difference found between the two Californian species, but our results show that extrapolating it to other parts of the world was a mistake. Remarkably, Heyning & Perrin [6] noticed not only that modal RL/ZW ratios were higher in South Africa, but also that vertebrae counts differed between long-beaked common dolphins from California (77–80 vertebrae) and South Africa (72–76 vertebrae), but chose to ignore that difference and focus, instead, on the RL/ZW ratio. Once the distinction of the two species in California was established both by good morphometric and genetic works, and the synonymy of *D*. *bairdii* and *D*. *capensis* had been wrongly proposed by van Bree & Purves [12], *D*. *delphis* and *D*. *capensis* became the two accepted species in the genus, with the RL/ZW ratio as the sole diagnostic character between them. This was used by researchers to identify common dolphins worldwide, even when genetic data consistently indicated that short-beaked and long-beaked common dolphins from the Atlantic did not form reciprocally monophyletic groups [21,22]. Interestingly, even though the latter authors did not draw any taxonomic conclusions on their work, which was based on both nuclear and mitochondrial markers, they hint at the possible non validity of *D*. *capensis*, stating that “the presently recognized long-beaked common dolphin species (*Delphinus capensis*) may prove to be invalid”, but at the same time that “it seems unlikely, despite their close genetic relationship, that all ecologically and morphologically distinct *Delphinus* populations belong to the same species”. One of the reasons for their reluctance to reject the validity of *D*. *capensis* may have been their limited sampling of SW Atlantic dolphins (N = 7). When we used a larger sample size (N = 37 new samples), including an area where long-beaked and short-beaked dolphins live in sympatry, it became clear that the two morphotypes did not correspond to genetically distinct groups (Fig 3) and are, probably, the result of phenotypic plasticity.

The difference in rostral length that distinguishes the two morphotypes seems to be related to niche partitioning, rather than speciation. Recently, Pinela *et al*. [36] found that short and long-beaked common dolphins from Mauritania had different isotopic signatures, which seem to reflect different feeding habits. The morphology of the rostrum is highly correlated with feeding specialisation, and some authors have suggested that as an explanation for convergence on the long-beaked morphotype [18,21]. According to Pinela *et al*. [36], in Mauritania longer beaks would correspond to feeding either in a higher trophic level or in more offshore habitats, in comparison to shorter beaks. Correlation between rostrum length and distance to the coast has been observed in many localities where the two morphotypes occur sympatrically, although usually the opposite pattern has been reported (longer beaks associated with shallower waters) [6,14,16].

Because mitochondrial DNA is inherited as a single locus, it is more susceptible to the confounding effects of stochastic lineage sorting and introgression, and may not depict the true evolutionary history, especially in the case of recent radiations [39–41]. Thus, the cytochrome b tree may not correspond to the species tree. The paraphyly observed in our trees could result from ancestral shared polymorphisms between very recently diverged species, or to historical and/or recurrent hybridisation between them. Studies have been able to detect shallow divergence of delphinid species using cytochrome b data [42–45], but this gene could simply be uninformative for *Delphinus*. However, the differentiation of long-beaked common dolphins from NE Pac shown in the cytochrome b tree seems to contradict this hypothesis. In any case, treatment of the *Delphinus* issue will probably benefit from including other mitochondrial genes (e.g. [46]), if not the complete mitogenome.

In addition, caution should be taken when considering mitochondrial data alone, especially a single locus. But with *Delphinus*, other genetic markers also do not support the existence of two cosmopolitan species. The studies by Natoli *et al*. [21] and Amaral *et al*. [22] used different genetic markers and slightly different geographic sampling, with similar results, which are also similar to ours. Nevertheless, our main conclusions are quite dissimilar, owing to different interpretation of data, especially in the case of Amaral *et al*. [22]. It is interesting that, even though these latter authors could not recover a statistically supported phylogeny of *Delphinus* species through Bayesian coalescence reconstruction based on a reasonable set of gene loci (one mitochondrial and five nuclear ones), they preferred to argue for the existence of several speciation events that might have taken place around the world. The reason for the lack of reciprocal monophyly would be incomplete lineage sorting (shared ancestral polymorphisms) and possibly extensive introgression, both resulting from a recent radiation of *Delphinus* (although the analytical method they used is theoretically adequate to solve genealogical relationships in such scenarios, [47]). We agree that incomplete lineage sorting and hybridisation may be important phenomena in the recent evolution of *Delphinus*. However, we consider that if they are so pervasive as to obliterate any phylogenetic signal, *D*. *delphis* and *D*. *capensis* should still be considered a single species.

Considering the worldwide distribution of common dolphins, the only genetic differentiation possibly strong enough to imply specific status would be that of long-beaked common dolphins from the NE Pacific. They have been considered recently as a highly differentiated population of *Delphinus capensis* [21,22], but since that species is not valid, they may in fact correspond to the *Delphinus bairdii* of Dall [48]. Two studies before Heyning & Perrin [6] argued in favour of two common dolphin species in the NE Pacific [37,49]. Van Bree & Purves [12] recognised the two morphotypes in California, but believed that intermediate types found in other parts of the world indicated they all belonged to *D*. *delphis*. Taking into consideration that long-beaked common dolphins from the NE Pacific are genetically differentiated from all other common dolphins ([11,21,22,38] and this study), the revalidation of *Delphinus bairdii* may be justified.

Our analyses also affect the currently accepted subspecies of *D*. *capensis*. Despite the very few sequences available to date, genetic data do not support the validity of the subspecies *Delphinus capensis tropicalis* van Bree, 1971 [5,50] (which, in any case, would be a subspecies of *D*. *delphis*, considering that *D*. *capensis* is invalid). The *tropicalis*-form is genetically divergent from other common dolphin populations (Table S1 of [22], but the paraphyly observed in phylogenetic trees ([22] and this study) argues against considering it a taxonomically valid entity. However, subspecific differentiation is not necessarily expected to result in monophyly, due to the very shallow divergence, which leads to incomplete lineage sorting and facilitates hybridisation. Another possibility would be slow lineage sorting due to very large effective population sizes–a likely scenario for pelagic schooling dolphins such as *Delphinus* in the Indian Ocean. In this setting, genetic drift would not have been severe enough to result in monophyly. The morphological evidence provided by Amaha [15] may suffice to support the subspecies *D*. *delphis tropicalis*. In the case of common dolphins from the Black Sea (*Delphinus delphis ponticus* Barabash-Nikiforov, 1935 [51]), preliminary genetic data suggest that they differ from those from the Eastern Mediterranean (*D*. *delphis delphis*) [52], but more samples need to be analysed to clarify this issue. The subspecific status of the *tropicalis*-form and of Black Sea common dolphins should be further investigated with the inclusion of more samples, genetic markers with higher resolution and phylogeographic analyses.

The issue about *Delphinus* species and subspecies should continue to receive attention in the coming years. Future studies using genetic data with increased resolution, including mitogenomes and a larger coverage of nuclear genomes from several specimens, should provide further clarity for the taxonomy of this genus. For example, recent mitogenomic data from 139 killer whales (*Orcinus orca*) provided unprecedented support for the genetic differentiation of ecotypes [53], which was corroborated by nuclear phylogenomic analyses using RAD-sequencing data [54]. Those results may lead to revision of the currently monotypic genus *Orcinus*. Analyses such as these are becoming more accessible due to next-generation sequencing technologies, and will likely be a promising avenue for future research on the *Delphinus* issue.

### Implications to conservation

The short-beaked common dolphin *D*. *delphis* is currently listed by IUCN as “least concern”, due to its abundance and widespread distribution [55]. Abundance and mortality data are available for the NW and NE Atlantic, NE Pacific, Mediterranean (reviewed by [55]), Central Atlantic [56] and SE Pacific [57,58] but no information exist for populations in the South Atlantic (except for South Africa and Gabon, [59,60]).

IUCN recognises *D*. *capensis* and its two subspecies (*D*. *c*. *capensis* and *D*. *c*. *tropicalis*), all listed as “data deficient”. The only available data on abundance and mortality are for the NE Pacific [61,62], which, according to genetic data, may be a distinct species (*D*. *bairdii*). If that conclusion is confirmed by other analyses, long-beaked common dolphins from the NE Pacific will merit a conservation status due to its endemicism. Given our results, all other long-beaked common dolphin populations in fact belong to *D*. *delphis*. The *tropicalis-*form of the Indian Ocean would be a subspecies of *D*. *delphis*.

Due to the availability of a single cytochrome b sequence, we could not draw conclusions about the subspecific status of the Black Sea common dolphin (*D*. *delphis ponticus*), which is considered “vulnerable” by IUCN.

## Conclusions

We tested the two species hypothesis by assigning sympatric SW Atlantic specimens of differing morphotypes to either *Delphinus* species according to the rostral index of Heyning & Perrin [6], and verifying if they corresponded to *D*. *delphis* or *D*. *capensis* using the cytochrome b gene, as outlined by Rosel *et al*. [11]. Using an extensive dataset with sequences from several localities worldwide, our test failed to find support for the two species globally. However, genetic data confirmed that long-beaked common dolphins from the NE Pacific may be a distinct species.

Given our results and those from previous studies, *D*. *capensis* is invalid. Therefore, we recommend the use of *D*. *capensis* to refer to long-beaked forms globally be discontinued. We also suggest that the name *D*. *bairdii* Dall, 1873 be used for long-beaked common dolphins restricted to the NE Pacific until further, more comprehensive analyses can be conducted.

## Supporting Information

S1 Fig Preliminary phylogenetic tree (NJ/ML/BI) of cytochrome b sequences from all delphinid species, including common dolphin haplotypes observed in this study. Values in nodes correspond to bootstrap, aLRT and posterior probabilities (NJ/ML/BI, respectively) > 50%. Single values refer to NJ bootstraps.
(DOC)

S1 Table GenBank accession number of the haplotype sequences used in this study, and localities where they were collected. (DOCX)
