# Correction: Molecular and Morphological Differentiation of Common Dolphins (*Delphinus* sp.) in the Southwestern Atlantic: Testing the Two Species Hypothesis in Sympatry

**DOI:** 10.1371/journal.pone.0145354

**Published:** 2015-12-14

**Authors:** Haydée A. Cunha, Rocio Loizaga de Castro, Eduardo R. Secchi, Enrique A. Crespo, José Lailson-Brito, Alexandre F. Azevedo, Cristiano Lazoski, Antonio M. Solé-Cava

There is an error in affiliation 3 for author Eduardo R. Secchi. Affiliation 3 should be: Laboratório de Ecologia e Conservação da Megafauna Marinha (EcoMega), Instituto de Oceanografia, Universidade Federal do Rio Grande, Rio Grande, Rio Grande do Sul, Brazil.
