# Supplementary material for: Molecular and Morphological Differentiation of Common Dolphins (Delphinus sp.) in the Southwestern Atlantic: Testing the Two Species Hypothesis in Sympatry
Source: PLoS One. 2015 Nov 11;10(11):e0140251. doi: 10.1371/journal.pone.0140251 (PMC4641715; doi:10.1371/journal.pone.0140251)
Supplement: S1 Fig — Values in nodes correspond to bootstrap, aLRT and posterior probabilities (NJ/ML/BI, respectively) > 50%. Single values refer to NJ bootstraps. (DOC) [file pone.0140251.s001.doc]

*Sousa chinensis*

*S. chinensis*

*Stenella attenuata*

*S.attenuata*

*S. attenuata*

*S.attenuata*

*T.* *australis*

*Lagenodelphis hosei*

*L. hosei*

*Stenella longirostris*

*S. longirostris*

*S. longirostris*

*S. longirostris*

*S.longirostris*

*S.longirostris*

*S.longirostris*

*Lissodelphis peronii*

*Lissodelphis* borealis

*Lagenorhynchus obscurus*

*Lagenorhynchus obliquidens*

*Lagenorhynchus cruciger*

*Lagenorhynchus australis*

*Cephalorhynchus heavisidii*

*Cephalorhynchus hectori1*

*Cephalorhynchus eutropia*

*Cephalorhynchus comersonii*

*Steno bredanensis*

*S. bredanensis*

*Sotalia guianensis*

*Sotalia fluviatilis*

*S.* *fluviatilis*

*S. fluviatilis*

*Lagenorhynchus albirostris*

*L.albirostris*

*Orcinus orca*

*O.orca*

*Orcaella brevirostris*

*O. brevirostris*

*O.brevirostris*

*Grampus griseus*

*G. griseus*

*Pseudorca crassidens*

*Feresa attenuata*

*Peponocephala electra*

*Globicephala melas*

*G. melas*

*Globicephala macrorhynchus*

*G. macrorhynchus*

*Lagenorhynchus acutus*

*Pontoporia blainvillei*

99

100

100

100

100

79

100

100

100

100

100

100

99

99

99

98

99

92

99

95

88

96

100

100

100

100

95

99

99

96

95

95

100

94/94/100

84

98

95/94/100

94

87

79/81/100

99/99/100

95/81/100

89

0.02

Hap46 (ARG)

Hap9 (RS, ARG)

*Delphinus delphis* (NE Atl)

Hap10 (RJ, SP, ARG, NE Atl)

Hap11 (RJ)

Hap49 (ARG)

Hap44 (RJ)

Hap48 (ARG)

Hap50 (ARG)

*Delphinus delphis* (Black Sea)

*D. delphis* (NE Atl)

Hap3 (RS, ARG)

Hap45 (ARG)

Hap7 (RS)

Hap52 (ARG)

*D. delphis* (NE Atl)

*D. delphis* (NE Atl)

*D. delphis* (NE Atl)

Hap8 (RS)

*D. delphis* (NE Atl)

*D. delphis* (NE Atl)

*D. capensis* (China)

*Delphinus capensis* (Pacific Ocean)

*D. capensis* (Pacific Ocean)

Hap47 (ARG)

Hap51 (ARG)

Hap5 (RS)

Hap4 (RS)

*Delphinus delphis* (Pacific Ocean)

*Delphinus tropicalis* (Indian Ocean)

*D. delphis* (NE Atl)

*D. delphis* (NE Atl)

*D. delphis* (NE Atl)

*D. delphis* (NE Atl)

Hap6 (ARG)

*Stenella clymene*

RS9

*Stenella coeruleoalba*

*Stenella coeruleoalba*

*Tursiops aduncus*

*T. aduncus*

*Stenella frontalis*

*S. frontalis*

*Tursiops truncatus*

*T. truncatus*

*T.truncatus*

*T. truncatus*

*T. truncatus*
