# Supplementary material for: Molecular and Morphological Differentiation of Common Dolphins (Delphinus sp.) in the Southwestern Atlantic: Testing the Two Species Hypothesis in Sympatry
Source: PLoS One. 2015 Nov 11;10(11):e0140251. doi: 10.1371/journal.pone.0140251 (PMC4641715; doi:10.1371/journal.pone.0140251)
Supplement: S1 Table — (DOCX) [file pone.0140251.s002.docx]

| **Haplotype** | **GenBank #** | **Locality** |
| --- | --- | --- |
| H1 | DQ378153 | NE Atlantic |
| H2 | AF084085 | NE Pacific (Sb) |
| H3 | KC297763 | NE, SE, SW, CE Atlantic, SW Pacific, Black Sea |
| H4 | KC297720 | NE Pacific (Lb) |
| H5 | AF084088 | NE Pacific (Lb), NW Atlantic, Indian (*tropicalis*) |
| H6 | EU557094 | China |
| H7 | KC297711 | NE Pacific (Lb) |
| H8 | EF090636 | NE Atlantic |
| H9 | KC297760 | NE, CE Atlantic |
| H10 | KC297749 | NE, SE Atlantic |
| H11 | KC297743 | NE, NW, CE Atlantic |
| H12 | DQ378138 | NE Atlantic |
| H13 | DQ378140 | NE Atlantic |
| H14 | KC297725 | NE Atlantic |
| H15 | KC297723 | NE Atlantic |
| H16 | DQ378143 | NE Atlantic |
| H17 | KC297724 | NE Atlantic |
| H18 | KC297728 | NE, CE Atlantic, SW Pacific, Indian (*tropicalis*) |
| H19 | KC297721 | NE Atlantic |
| H20 | DQ378149 | NE Atlantic |
| H21 | DQ378150 | NE Atlantic |
| H22 | KC297765 | NE Atlantic |
| H23 | DQ378156 | NE Atlantic |
| H24 | DQ378158 | NE Atlantic |
| H25 | DQ378159 | NE Atlantic |
| H26 | DQ378160 | NE Atlantic |
| H27 | DQ378161 | NE Atlantic |
| H28 | DQ378163 | NE Atlantic |
| H29 | KC297742 | NE, NW Alantic |
| H30 | KM225661 | SW Atlantic |
| H31 | KM225662 | SW Atlantic |
| H32 | KM225663 | SW Atlantic |
| H33 | KM225664 | SW Atlantic |
| H34 | KM225665 | SW Atlantic |
| H35 | KC297729 | SW Pacific, SW Atlantic |
| H36 | KM225666 | SW Atlantic |
| H37 | KM225667 | SW Atlantic |
| H38 | KM225668 | SW Atlantic |
| H39 | KM225669 | SW Atlantic |
| H40 | KM225670 | SW Atlantic |
| H41 | KM225671 | SW Atlantic |
| H42 | JX264702 | SW Pacific, SW Atlantic |
| H43 | KM225672 | SW Atlantic |
| H44 | KC297733 | SW Pacific, SW Atlantic |
| H45 | JX264646 | SW Pacific, SW Atlantic |
| H46 | JX264631 | SW, NE Pacific, SW Atlantic |
| H47 | KM225673 | SW Atlantic |
| H48 | KC297757 | SE Atlantic |
| H49 | KC297755 | SE Atlantic |
| H50 | JX264666 | SW Pacific |
| H51 | JX264674 | SW Pacific |
| H52 | JX264673 | SW Pacific |
| H53 | JX264670 | SW Pacific |
| H54 | JX264668 | SW Pacific |
| H55 | KC297717 | NE Pacific (Lb) |
| H56 | KC297713 | NE Pacific (Lb) |
| H57 | KC297764 | CE Atlantic |
| H58 | KC297756 | SE Atlantic |
| H59 | KC297754 | SE Atlantic |
| H60 | KC297744 | SE Atlantic |
| H61 | KC297740 | SW Pacific |
| H62 | JX264675 | SW Pacific |
| H63 | JX264672 | SW Pacific |
| H64 | KC297730 | SW Pacific |
| H65 | JX264658 | SW Pacific |
| H66 | JX264622 | NE Pacific (Lb) |
| H67 | KC297712 | NE Pacific (Lb) |
| H68 | JX264700 | SW Pacific |
| H69 | JX264698 | SW Pacific |
| H70 | JX264616 | SW Pacific |
| H71 | JX264694 | SW Pacific |
| H72 | JX264692 | SW Pacific |
| H73 | JX264690 | SW Pacific |
| H74 | JX264688 | SW Pacific |
| H75 | JX264686 | SW Pacific |
| H76 | JX264682 | SW Pacific |
| H77 | JX264680 | SW Pacific |
| H78 | JX264678 | SW Pacific |
| H79 | JX264676 | SW Pacific |
| H80 | JX264664 | SW Pacific |
| H81 | JX264662 | SW Pacific |
| H82 | JX264660 | SW Pacific |
| H83 | JX264656 | SW Pacific |
| H84 | JX264654 | SW Pacific |
| H85 | JX264652 | SW, NE Pacific |
| H86 | JX264650 | SW Pacific |
| H87 | JX264648 | SW Pacific |
| H88 | JX264644 | SW Pacific |
| H89 | JX264640 | SW Pacific |
| H90 | JX264638 | SW Pacific |
| H91 | JX264636 | SW Pacific |
| H92 | JX264634 | NE Pacific (Sb) |
| H93 | JX264632 | NE Pacific (Sb) |
| H94 | JX264630 | NE Pacific (Sb) |
| H95 | JX264628 | NE Pacific (Sb) |
| H96 | JX264626 | NE Pacific (Sb) |
| H97 | JX264622 | NE Pacific (Sb) |
| H98 | JX264620 | NE Pacific (Sb) |
| H99 | JX264618 | NE Pacific (Sb) |
| H100 | JX264616 | NE Pacific (Sb) |
| H101 | JX264614 | NE Pacific (Sb) |
| H102 | JX264612 | NE Pacific (Sb) |
| H103 | JX264610 | NW Atlantic |
| H104 | JX264608 | NW Atlantic |
| H105 | JX264604 | NW Atlantic |
| H106 | JX264602 | NW Atlantic |
| H107 | JX264600 | NW Atlantic |
| H108 | JX264598 | NW Atlantic |
| H109 | JX264594 | CE Atlantic |
| H110 | JX264576 | NE Atlantic |
| H111 | JX265572 | Indian (*tropicalis*) |
| H112 | JX264570 | Indian (*tropicalis*) |
| H113 | JX264568 | Indian (*tropicalis*) |
| H114 | JX264701 | SW Pacific |
| H115 | JX264699 | SW Pacific |
| H116 | JX264697 | SW Pacific |
| H117 | JX264495 | SW Pacific |
| H118 | JX264693 | SW Pacific |
| H119 | JX264691 | SW Pacific |
| H120 | JX264689 | SW Pacific |
| H121 | JX264687 | SW Pacific |
| H122 | JX264685 | SW Pacific |
| H123 | JX264683 | SW Pacific |
| H124 | JX264681 | SW Pacific |
| H125 | JX264679 | SW Pacific |
| H126 | JX264661 | SW Pacific |
| H127 | JX264659 | SW Pacific |
| H128 | JX264657 | SW Pacific |
| H129 | JX264655 | SW Pacific |
| H130 | JX264653 | SW Pacific |
| H131 | JX264649 | SW Pacific |
| H132 | JX264647 | SW Pacific |
| H133 | JX264645 | SW Pacific |
| H134 | JX264643 | SW Pacific |
| H135 | JX264641 | SW Pacific |
| H136 | JX264639 | SW Pacific |
| H137 | JX264637 | SW Pacific |
| H138 | JX264635 | SW Pacific |
| H139 | JX264633 | NE Pacific (Sb) |
| H140 | JX264629 | NE Pacific (Sb) |
| H141 | JX264627 | NE Pacific (Sb) |
| H142 | JX264625 | NE Pacific (Sb) |
| H143 | JX264623 | NE Pacific (Sb) |
| H144 | JX264621 | NE Pacific (Sb) |
| H145 | JX264619 | NE Pacific (Sb) |
| H146 | JX264617 | NE Pacific (Sb) |
| H147 | JX264615 | NE Pacific (Sb) |
| H148 | JX264613 | NE Pacific (Sb) |
| H149 | JX264611 | NW Atlantic |
| H150 | JX264609 | NW Atlantic |
| H151 | JX264605 | NW Atlantic |
| H152 | JX264601 | NW Atlantic |
| H153 | JX264599 | NW Atlantic |
| H154 | JX264597 | CE Atlantic |
